# Stress and reward in the maternal brain of mothers with borderline personality disorder: a script-based fMRI study

**DOI:** 10.1007/s00406-023-01634-6

**Published:** 2023-06-24

**Authors:** Isabella Schneider, Sabine C. Herpertz, Kai Ueltzhöffer, Corinne Neukel

**Affiliations:** 1 Department of General Psychiatry, Center for Psychosocial Medicine, Heidelberg University, Voßstr. 4, 69115 Heidelberg, Germany; 2 European Molecular Biology Laboratory, Genome Biology Unit, Meyerhofstr. 1, 69117 Heidelberg, Germany

**Keywords:** Insula, ACC, Mother–child interaction, Salience, Stress, Reward

## Abstract

Borderline personality disorder (BPD) is associated with altered neural activity in regions of salience and emotion regulation. An exaggerated sensitization to emotionally salient situations, increased experience of emotions, and dysfunctional regulative abilities could be reasons for increased distress also during parenting. Mothers with BPD tend to have less reciprocal mother–child interactions (MCI) and reveal altered cortisol and oxytocin reactivity in the interaction with their child, which could indicate altered processing of stress and reward. Here, we studied underlying neural mechanisms of disrupted MCI in BPD. Twenty-five mothers with BPD and 28 healthy mothers participated in a script-driven imagery functional magnetic resonance imaging (fMRI)-paradigm. Scripts described stressful or rewarding MCI with the own child, or situations in which the mother was alone. Mothers with BPD showed larger activities in the bilateral insula and anterior cingulate cortex (ACC) compared to healthy mothers during the imagination of MCI and non-MCI. Already in the precursory phase while listening to the scripts, a similar pattern emerged with stronger activity in the left anterior insula (AINS), but not in the ACC. This AINS activity correlated negatively with the quality of real-life MCI for mothers with BPD. Mothers with BPD reported lower affect and higher arousal. An exaggerated sensitization to different, emotionally salient situations together with dysfunctional emotion regulation abilities, as reflected by increased insula and ACC activity, might hinder sensitive maternal behavior in mothers with BPD. These results underline the importance for psychotherapeutic interventions to improve emotional hyperarousal and emotion regulation in patients with BPD, especially in affected mothers caring for young children.

**Supplementary Information:**

The online version contains supplementary material available at 10.1007/s00406-023-01634-6.

## Introduction

Borderline personality disorder (BPD) is characterized by instability in self, emotion dysregulation, and dysfunctional interactional processes. Altered facial emotion processing, threat hypersensitivity, and increased rejection sensitivity have been described as possible underlying impairments [1–3] and may lead to particular challenges when individuals with BPD are parents and interact with their children. Mothers with BPD seem to experience increased levels of parenting distress in self-report and feel less competent in their parenting [4]. They tend to show more intrusive behaviors, less reciprocal mother–child interactions on a dyadic level and reveal altered oxytocin and cortisol reactivity, which could indicate a lack of reward and of relief of stress in the interaction with their child [5].

More and more research tries to understand the neurobiological underpinnings of the disorder, bringing up the theory of fronto-limbic imbalance [6, 7]. The general model of cognitive control of emotion (MCCE) aims to explain emotion regulation—a central topic in BPD—by integrating knowledge on behavior and experience, information processing, and neural systems [8]. It describes mechanisms involved in generating an emotion (affect system) as well as mechanisms involved in cognitive control, and, thus, can help to understand neural alterations in BPD. According to the MCCE, the amygdala, anterior insula (AINS), ventral striatum (VS) and orbitofrontal cortex are involved in emotion generation [8]. These areas—especially the amygdala and insula [9, 10]—have often been reported to show increased activity in BPD in various contexts, especially in between-groups contrasts for negative—neutral experimental conditions [10, 11], but also during a social inclusion condition as compared to a passive watching condition [9]. This could relate to an emotional over-involvement, increased processing and augmented negative appraisal of emotional stimuli in BPD in areas involved in emotion regulation (MCCE) [10, 11] and the symptom of intense and instable emotions. Additionally, especially the AINS and the dorsal anterior cingulate cortex (ACC) are also involved in the salience network, which detects, classifies and re-evaluates salient stimuli out of the broad range of information (one perceives) and which could play an important role in emotion regulation in BPD [12, 13]. An exaggerated sensitization to emotionally salient situations, an increased experience of emotions, and reduced or dysfunctional emotion regulation abilities could be reasons for increased experienced distress in individuals with BPD [13, 14].

Furthermore, the MCCE describes the ACC, the dorsolateral, ventrolateral, and dorsomedial prefrontal cortex (PFC) as important regions for emotion processing, regulation and cognitive control with modulating effects on the affect system [8]. In BPD, meta-analyses reported enhanced activity in the ventrolateral prefrontal cortex (PFC) [11], reduced activity in dorsolateral prefrontal areas [7], and heterogeneous results with hypo- and hyperactivations in comparison with healthy controls probably depending on the study design and stimuli used for the ACC [6, 10]. Individuals with BPD also seem to have a reduced striatal neural response to reward and loss anticipation in response to monetary rewards compared to healthy controls [15]. Such a result could indicate alterations in the neural processing of reward and could also affect social interactions, if similar patterns apply to social rewards.

The concept of the “maternal brain” highlights neural systems—that have also been shown to be altered in BPD—including (among others) emotion processing and salience of social cues (amygdala, insula, ACC), emotional and cognitive control (ventrolateral and dorsolateral PFC, ACC), and reward and motivation (VS), which are described to be critical for parenting [16, 17]. These systems might serve to increase neural and behavioral sensitivity to child cues, as well as self- and co-regulative processes in the context of (distressful) interactions in healthy mothers [17]. For emotion processing and control, the maternal brain concept relates well to the MCCE as effective emotion regulation with a well-regulated prefrontal and cingulate control system, which can modulate activity in (para-)limbic affect systems, is highly important for sensitive parenting. Healthy mothers showing intrusive maternal behaviors demonstrated an overactivation of the amygdala, which has been interpreted as an explanation for non-matched excessive maternal behavior [18]. An excessive activity in the AINS can lead to high maternal distress, an emotional over-involvement in the child’s distress, and reduced maternal abilities to co-regulate the child’s distress [17, 19]. Stress in turn can negatively affect maternal response behavior and emotion regulation capacity and impacts the maternal brain adaptation [17]. Reward-related mechanisms seem to be essential underlying factors in maternal behavior and motivate mothers to contact and care for their child [16]. Functional magnetic resonance imaging (fMRI) studies have shown that sounds, pictures, or videos of the child activate areas in the reward system of healthy mothers [20–22]. Stronger activations of the nucleus accumbens were shown in mothers with sensitive maternal behavior compared to mothers with intrusive maternal behavior [18]. Up to now, brain activity in mothers with BPD has not been studied.

Here, we analyzed neural activity in significant areas of the maternal brain with a focus on stress and reward in mothers with BPD compared to healthy mothers using a script-driven imagination-based fMRI paradigm. Scripts described stressful or rewarding mother–child interactions, or situations in which the mother was alone. We expected altered activity in areas of (1) emotion processing and salience (amygdala, insula, ACC), (2) emotional and cognitive control (ventrolateral and dorsolateral PFC, ACC), and (3) reward and motivation (ventral striatum) in mothers with BPD compared to healthy mothers. These alterations could be specifically pronounced for mother–child interactions. We further investigated in an explorative approach neural activity in the different phases of stimulation (audio phase, imagination phase), as well as associations with the quality of real-life mother–child interactions.

## Materials and methods

### Participants

Twenty-five mothers with a current diagnosis of BPD (M_number of BPD symptoms_ = 6.4 ± 1.1; M_age_ = 31.2 ± 7.0 years) and 28 healthy mothers without a current or lifetime psychiatric diagnosis (HC; M_age_ = 31.9 ± 5.0 years; t_51_ = − 0.44, *p* = 0.663) were included in the study. Mothers did not differ in intelligence estimation (BPD: M = 26.4 ± 9.8; HC: M = 29.5 ± 8.9; t_51_ = − 1.21; *p* = 0.233). The participants’ children were between 18- and 36-months old (BPD: M_age_ = 27.2 ± 7.0 months; HC: M_age_ = 27.4 ± 6.0 months; t_51_ = − 0.13, *p* = 0.897; BPD/HC: 12/13 girls, 13/15 boys; Χ^2^_1_ = 0.01, *p* = 0.909); please also see Table 1. General exclusion criteria were age < 18 and > 50 years, MRI contraindications, neurological disorders, organic brain damage, severe medical illness, alcohol or drug (nicotine excluded) dependence over the last 24 months, left-handedness, mother and child not living together, pregnancy, and breast-feeding. Comorbidities in mothers with BPD were as follows: posttraumatic stress disorder: *n* = 6, major depression: *n* = 9, anxiety disorder: *n* = 6, obsessive–compulsive disorder: *n* = 1. Six mothers with BPD took psychotropic medication (SSRI: *n* = 4; SSNRI: *n* = 1; Ritalin: *n* = 1). Originally, 26 mothers with BPD and 30 healthy mothers were assessed; however, one mother with BPD and two healthy mothers had to be excluded due to excessive head motion (BPD, HC) and a brain lesion (HC). Participants were screened via telephone and participated in a diagnostic interview and a behavioral observation [5].

**Table 1:** Descriptive data for group characteristics and behavioral data of mothers with BPD and healthy mothers (HC)

	BPD		HC		F/t/U_df_	*p*
	M	SD	M	SD		
Age	31.2	7.0	31.9	5.0	− 0.44_51_	0.663
Age child	27.2	7.0	27.4	6.0	− 0.13_51_	0.897
Intelligence	26.4	9.8	29.5	8.9	− 1.21_51_	0.233
CIB-score	2.9	0.8	3.5	0.4	442.5_51.16_	0.009
Affect ratings	3.2	0.3	3.4	0.4	5.24_1,51_	0.026
rMCI	4.0	0.7	4.5	0.4	− 3.39_51_	< 0.050*
sMCI	2.4	0.9	2.2	0.7	0.88_51_	> 0.050
Non-MCI	3.2	0.5	3.6	0.6	− 2.44_51_	> 0.050
Arousal ratings	2.2	0.4	1.8	0.5	10.25_1,51_	0.002
rMCI	1.6	0.5	1.3	0.5	1.86_51_	< 0.050*
sMCI	3.2	0.7	2.6	0.6	3.26_51_	< 0.050*
Non-MCI	1.8	0.6	1.4	0.7	2.35_51_	< 0.050*
Rating of vividness	4.6	0.3	4.7	0.3	− 0.56_51_	0.576

*r/sMCI* rewarding/stressful mother–child interaction, *nonMCI* no mother–child interaction, *according to Dunn’ post hoc test

### Measures

We assessed current and lifetime axis I disorders using the International Neuropsychiatric Interview (M.I.N.I.; [23]) and BPD using the International Personality Disorder Examination (IPDE; [24]). Intelligence was estimated using the mini-q [25], a reliable and validated screening for cognitive abilities based on the reasoning test in English presented by Baddeley [26].

Mother–child interaction was assessed by behavioral observation in a 10-min free-play situation of a sub-sample (BPD: *n* = 22; HC: *n* = 28). Interactions were rated for maternal, child, and dyadic behavior using the well validated “Coding Interactive Behavior” manual (CIB [27]). Composite scores were computed for maternal sensitivity, intrusiveness, and limit setting, child involvement and withdrawal, as well as dyadic reciprocity and negative states. A CIB total score, which included maternal, child, and dyadic behavior (((maternal sensitivity + maternal limit setting + child involvement + dyadic reciprocity) /4) / ((maternal intrusiveness + child withdrawal + dyadic negative states) /3))), was used to judge the overall quality of the interaction (0 = low, 5 = high). Two coders were trained to 90% agreement and rated the videos blind to groups and all other information. Interrater reliability was at 95% (intraclass *r* = 0.95) based on 12 video-taped interactions (24%) which were rated separately by the two coders. More information on the method and data of a largely overlapping sample is available at [5].

### FMRI paradigm

**Fig. 1:**
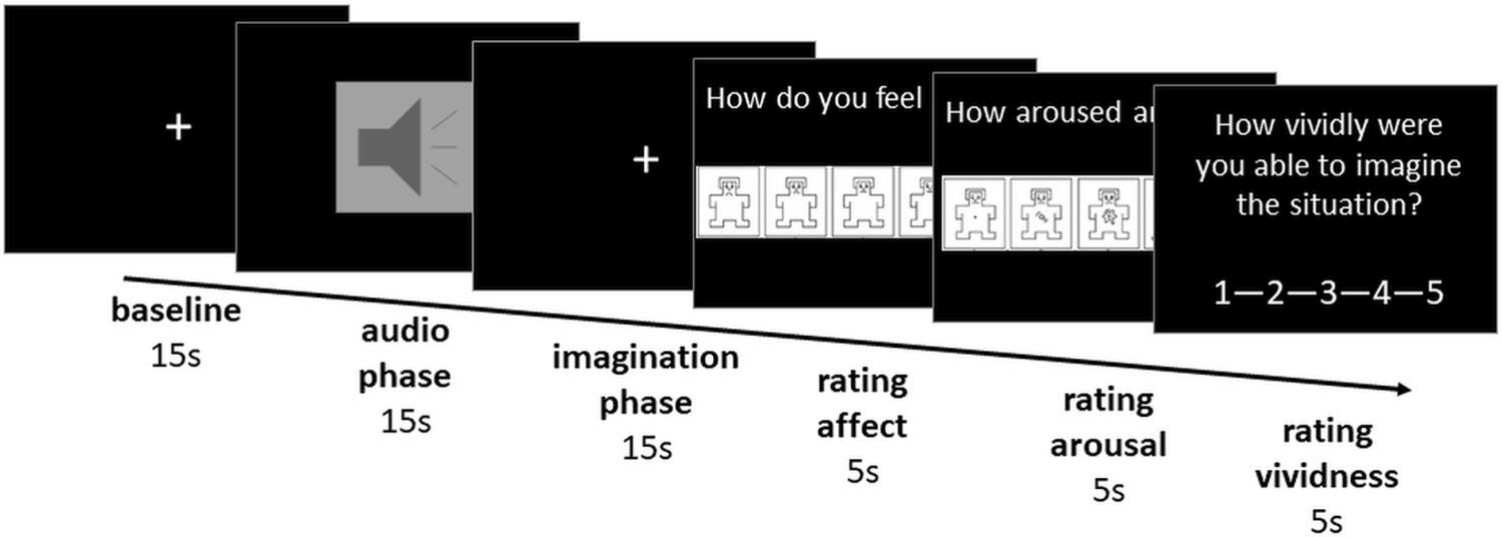
FMRI paradigm

We used a script-driven imagination-based paradigm based on Neukel et al. [19], which consisted of 24 acoustically presented pseudorandomized scripts depicting everyday situations presented in two sessions with a structural MR scan in between. At the beginning of the paradigm, mothers were instructed to listen to the scripts and imagine the situation as vividly as possible. After a 15 s baseline, a 15 s long script, read by a professional actress, was played [audio phase (AP)]. Then, mothers were asked to imagine the mother–child interaction for 15 s [imagination phase (IP)]. After that, participants rated their affect as a subjective evaluation of emotion (1 = negative to 5 = positive), arousal as a subjective evaluation of affective physiological reactions (1 = relaxed to 5 highly aroused), and the experienced vividness (1 = not at all to 5 = very good) in order to investigate associated affective states (Fig. 1). For the baseline, participants received the instruction to close their eyes, to lie still and think of nothing in particular. Phases were separated by audio signals. Scripts were divided into 8 scripts describing a rewarding mother–child interaction (rMCI; e.g., “I was outside and finally I am getting back to my daughter. When she sees me, she runs towards me and stretches her arms towards me. She smiles happily.”), 8 scripts describing a stressful mother–child interaction (sMCI; e.g., “I was outside and finally I am getting back to my daughter. When she sees me, she runs away from me and tries to hide. My daughter starts to cry and avoids my gaze.”) and 8 scripts of a comparable situation without the child (nonMCI; e.g., “I was outside and finally I am getting back home. I open the door and take off my shoes. I put the shoes next to the door. Then I take off my jacket.”). Scripts were all parallelized regarding the content, had the same length and same volume. For further information on validation of the stimuli and all scripts, please see online resource 1 and 2 in the supplementary materials.

### FMRI data acquisition

MRI scans were conducted using a 3 T whole body MR-scanner with a 32-channel head coil. After 22 participants (BPD: 11; HC: 11), the scanner was updated from a Magnetom TIM Trio to a Magnetom Prisma (Siemens). Thirty-three transverse slices (slice thickness: 3 mm) were acquired with a T2*-weighted echo-planar imaging sequence (TR: 2000 ms, TE: 30 ms; flip angle = 78°; field of view: 192 mm; in-plane resolution 3 × 3 mm). Structural images were recorded using a T1-weighted, sagittal oriented MPRAGE sequence with isotropic high-resolution (1 × 1 × 1 mm^3^). Prior to the paradigm, participants underwent a ~ 6 min resting-state scan. The paradigm lasted ~ 28 min. Participants were asked to move as little as possible and to follow the instructions described above (2.3).

### Data analyses

For demographic data, independent t-tests for continuous variables and Χ^2^-tests for categorical variables were used. Since CIB data were not normally distributed, a Mann–Whitney *U* test was used. The rating of vividness was used to document the successful completion of the task and was compared for possible group differences. Individual means were expected to be between 4 and 5, which was fulfilled by all participants. For ratings of affect and arousal, repeated-measure analyses of variance (rmANOVA) with group (BPD, HC) as between-subjects factor and condition (rMCI, sMCI, nonMCI) as within-subject factor with Dunn’s multiple comparisons with Bonferroni correction for multiple testing as post-hoc tests were used. Results were considered to be significant at *p* < 0.05. Partial eta squared (η_p_^2^) was used as a measure of effect sizes. Data analysis was performed using IBM SPSS Statistics 28.0 (IBM, Armonk, NY).

FMRI data were analyzed using Statistical Parametric Mapping 12 software (SPM 12; The Wellcome Centre for Human Neuroimaging, London, UK) implemented in MATLAB (R2017a, The MathWorks, Natick, MA, USA). Preprocessing included realignment, co-registration to the mean structural image, spatial normalization including segmentation, and smoothing with a 6 mm full width at half-maximum (FWHM) Gaussian filter. Motion parameters were assessed by calculating the framewise displacement (FD; [28]). Datasets with excessive head movements (FD > 3 mm) in more than 10% of the trials were excluded (*n* = 2). Otherwise, motion scrubbing was performed in order to eliminate functional volumes during which a FD > 3 mm occurred during the relevant phases from the statistical analysis (BPD: *n* = 3; HC: *n* = 2; maximum number of scrubbed scans: 2). The threshold of 3 mm was chosen with regard to the voxel size of the T2*-weighted echo-planar imaging sequence being 3 × 3 × 3 mm. On a first level, a general linear model was defined for each participant for the baseline, AP, IP, and rating phase as separate regressors for the three conditions (rMCI, sMCI, nonMCI), as well as six movement parameters. Each data set was high-pass filtered (cutoff 128 s) and first-order autoregressive processes corrected for temporal autocorrelations. Contrast images (rMCI-baseline, sMCI-baseline, nonMCI-baseline) were calculated for each participant for the AP and IP. On a second-level, a 2 × 3 full factorial design with group (BPD, HC) and condition (rMCI, sMCI, nonMCI) was used with type of scanner and use of medication as covariates of no interest. As our primary interest was the imagination phase, a first model was calculated for the IP. Since imagination already starts with the listening to the script, we also calculated a model for the audio phase to capture possible first reactions to the presented scripts. Due to our specific hypotheses on the maternal brain with a focus on stress and reward, we performed region-of-interest (ROI) analyses with a single mask including bilateral amygdala, insula, anterior cingulate cortex, ventral striatum, inferior frontal gyrus, and middle frontal gyrus. We used SPM small volume correction (SVC) of p_FWE_ < 0.05 on the respective *p* < 0.01 uncorrected whole-brain statistical maps. The regions for ROI analyses were chosen based on a review of Kim et al. [16], describing these areas as part of the “maternal brain,” and defined by the Neuromorphometrics atlas (Neuromorphometrics, Inc. (Somerville, MA, USA) http://Neuromorphometrics.com/, accessed on 02/05/21 under academic subscription). For comparison, results of a whole brain analysis can be found in the supplements (online resource 3).

Since we were also interested, if neural activity differences between groups were associated with real-life mother–child interaction, we analyzed Spearman’s correlations between significant neural clusters and the CIB-score. The MarsBaR Toolbox (0.44) [29] was used to extract parameter estimates form these clusters. Additionally, these parameter estimates were used to depict differences between AP and IP.

## Results

### Behavioral data

Mothers with BPD showed lower scores at the CIB-score (BPD: M = 2.9 ± 0.8; HC: M = 3.4 ± 0.4; U_51.16_ = 442.5, *p* = 0.009; Table 1). There was no significant group difference regarding the rating of vividness of imagination (t_51_ = − 0.56, *p* = 0.576).

**Fig. 2:**
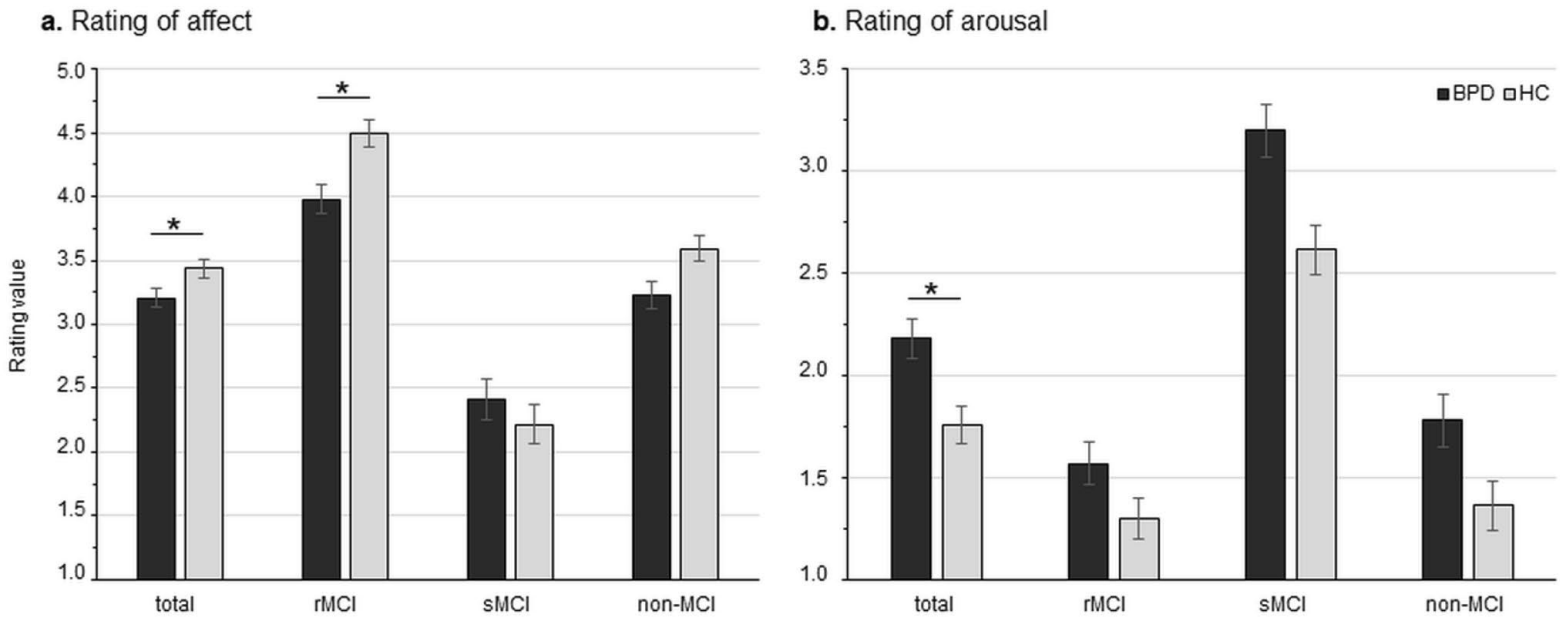
Rating of affect and arousal. **a** Significant group × condition interaction with lower ratings of affect in mothers with borderline personality (BPD) compared to healthy mothers (HC) in positive mother–child interactions (pMCI). **b** Significant group effect with higher ratings of affect in mothers with BPD compared to healthy mothers. *rMCI* rewarding mother–child interaction, *sMCI* stressful mother–child interaction, *nonMCI* no mother–child interaction. Significant comparisons are marked with an asterisk indicating *p* < 0.05 at the post-hoc test

Analyses of the affect ratings revealed a significant effect of condition (F_2,102_ = 115.60, *p* < 0.001, η_p_^2^ = 0.69) with highest affect ratings for rMCI (rMCI > nonMCI > sMCI), and a significant group effect (F_1,51_ = 5.24, *p* = 0.026, η_p_^2^ = 0.09) with lower affect ratings in BPD compared to HC. A significant interaction effect (F_2,102_ = 4.38, *p* = 0.023, η_p_^2^ = 0.08) showed lower affect ratings in the rMCI in BPD compared to HC but not for the sMCI or nonMCI (Fig. 2, Table 1).

Analyses of the arousal ratings revealed a significant effect of condition (F_2,102_ = 164.27, *p* < 0.001, η_p_^2^ = 0.76) with highest arousal during sMCI (sMCI > rMCI = nonMCI), and a significant group effect (F_1,51_ = 10.25, *p* = 0.002, η_p_^2^ = 0.17) with higher arousal ratings in BPD compared to HC (Fig. 2, Table 1). The interaction effect was not significant (F_2,102_ = 1.57, *p* = 0.217, η_p_^2^ = 0.03).

### ROI-analyses

**Fig. 3:**
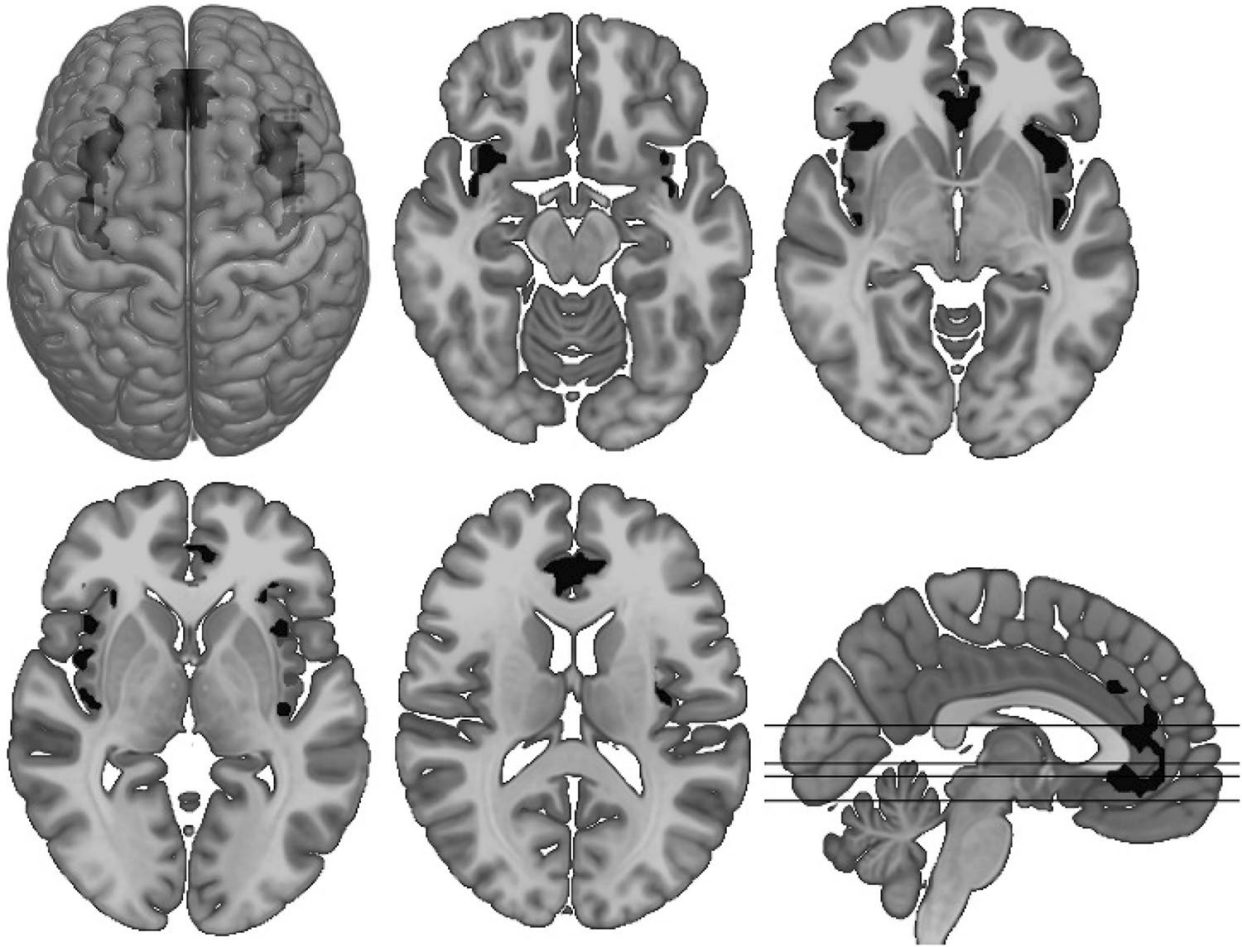
Significant neural clusters in the group comparison during the imagination phase. Mothers with borderline personality disorder show larger activity in bilateral insula and bilateral anterior cingulum compared to healthy mothers

In the imagination phase, analyses revealed a significant group difference with a stronger activation in the left AINS within a cluster extending to the left posterior insula (PINS), as well as a stronger activation in the right AINS extending to the right PINS in BPD compared to HC. Mothers with BPD also showed a stronger activation in the left pregenual ACC within a cluster extending from sub- to supragenual. A significant main effect of condition or group x condition interaction effect could not be found. Please see Table 2 and Fig. 3 for further information.

**Table 2:** Significant group differences between mothers with BPD and healthy mothers (HC) during the imagination phase

Contrast	Cluster size (k)	*T* value	*p* value peak	*p* value cluster FWE	Peak voxel MNI: x y z (mm)			Anatomical location of peak voxel	Anatomical location of cluster
BPD > HC	187	5.84	< 0.001	0.023	39	17	− 13	R AINS	R AINS, R PINS
	182	4.76	< 0.001	0.026	− 27	26	− 4	L AINS	L AINS, L PINS
	220	4.48	< 0.001	0.011	− 3	29	− 4	L ACC (pre)	L ACC (sup, sub), R ACC (pre, sup, sub)

*R* right, *L* left, *AINS* anterior insula, *PINS* posterior insula, *ACC* anterior cingulate cortex

The exploratory analysis of the audio phase revealed a significant group effect with a stronger activation in the left AINS in mothers with BPD compared to healthy mothers. Healthy mothers showed a significantly stronger activation in a cluster in the left ACC extending from pre- and subgenual to supragenual and to the right side in conditions with the child (rMCI, sMCI) compared to the condition without the child (nonMCI), but not in mothers with BPD. Please also see Table 3.

**Table 3:** Significant group differences between mother with BPD and healthy mothers (HC) during the audio phase

Contrast	Cluster size (k)	*T* value	*p* value peak	*p* value cluster FWE	Peak voxel MNI: x y z (mm)			Anatomical location of peak voxel	Anatomical location of cluster
BPD > HC	147	5.07	< 0.001	0.040	− 27	26	5	L AINS	L AINS
HC: r/sMCI > nonMCI	360	4.40	< 0.001	< 0.001	3	44	8	L ACC (pre)	L ACC (sup, sub); R ACC (pre, sub, sup)

*r/sMCI* rewarding/stressful mother–child interaction, *nonMCI* no mother–child interaction, *R* right, *L* left, *AINS* anterior insula, *ACC* anterior cingulate cortex

### Associations between fMRI and mother–child interaction

**Fig. 4:**
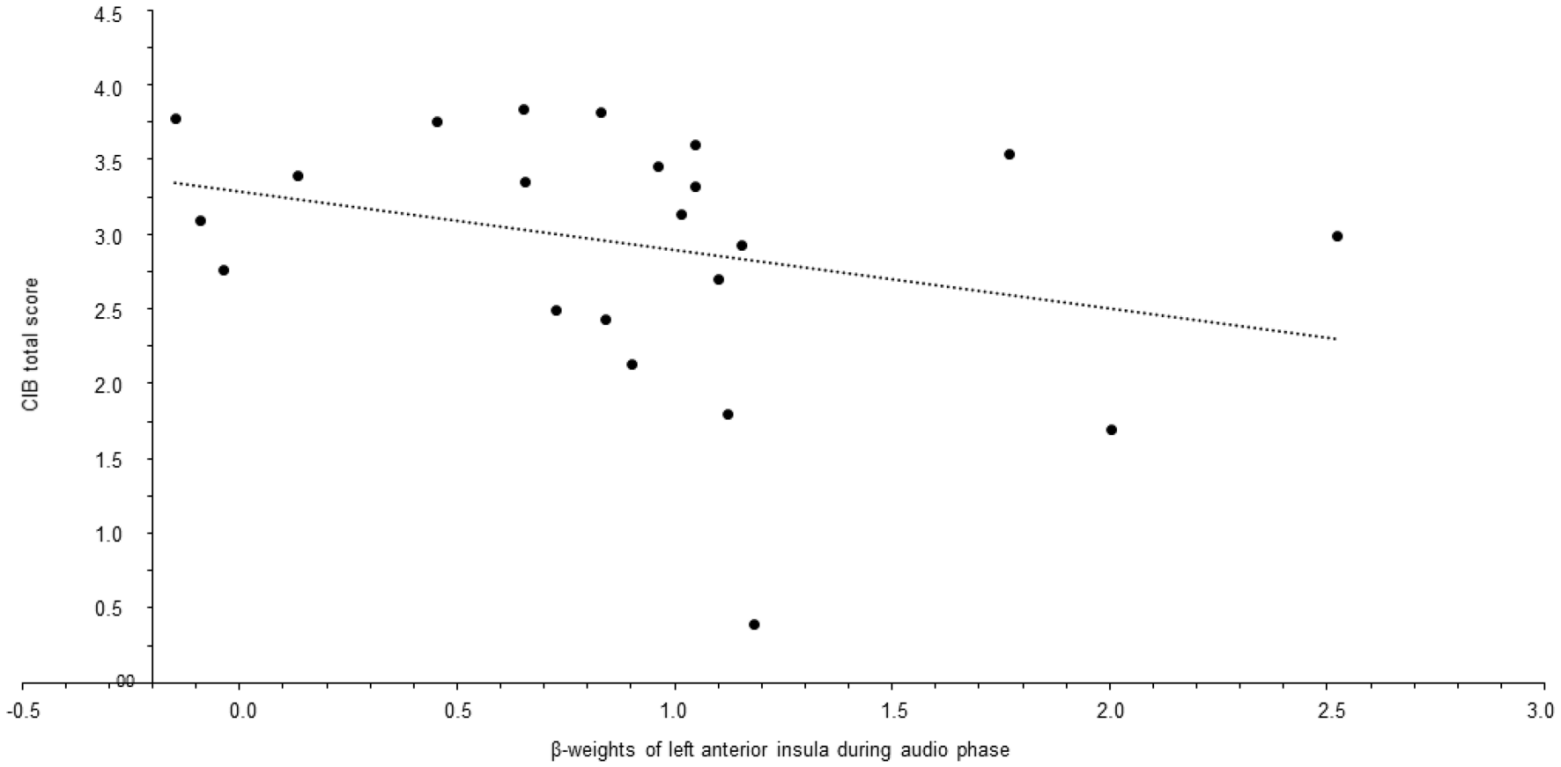
Associations between fMRI and mother–child interaction. Scatter plot of CIB-scores and β-weights of left anterior insula during audio phase for mothers with BPD

There was no significant correlation of clusters with the CIB-score during the imagination phase in mothers with BPD or in healthy mothers. During the audio phase, stronger activations in the left AINS correlated with lower values in the CIB-score in mothers with BPD (*r* = − 0.44, *p* = 0.042), but not in healthy mothers (*r* = 0.01, *p* = 0.967), who did not show much variance in the CIB-score (see also Fig. 4).

## Discussion

In the present study, we investigated neural activities in regions of the so-called maternal brain with a focus on stress and reward in a sample of mothers with BPD that has not been studied before. Mothers with BPD expressed a hyperactivation of the bilateral insula and ACC compared to healthy mothers during the imagination of stressful and rewarding mother–child interactions and non-mother–child-interactions (being with oneself). Additionally, during the audio phase, mothers with BPD showed stronger activity in the left AINS, but not in the ACC, compared to healthy mothers. Activity in the left AINS correlated negatively with the quality of real-life mother–child interactions in mothers with BPD. Interestingly, healthy mothers but not mothers with BPD showed increased activation in the ACC in response to listening to scripts describing an interaction with their child compared to nonMCI scripts. Mothers with BPD reported significantly lower affect, especially in the rMCI condition, and higher arousal during imagination of the scripts.

Firstly, the subjective ratings of affect and arousal underline that the experimental manipulation aiming at inducing emotional states occurring in mother–child interactions, worked: In line with the validation of the scripts (see online resource 1 in the supplement), rMCI elicited the highest (i.e., most positive) affect ratings and sMCI elicited the lowest affect (i.e., most negative) ratings overall, while sMCI elicited the highest arousal ratings across all participants. Interestingly, mothers with BPD reported lower affect ratings in response to rMCI scripts than healthy mothers, indicating group differences in emotional perception of rMCI that are in line with previous results, showing that patients with BPD rate the intensity of happiness in happy faces lower [30] and report less positive emotion (amusement, affection, contentment; [31]) than healthy controls. Regarding the arousal ratings, a significant group effect indicated higher arousal in mothers with BPD than in healthy mothers across all conditions.

As expected, we found altered neural activity in regions involved in emotion processing and salience, as well as emotional and cognitive control in response to everyday situations in mothers with BPD. As these alterations were not more pronounced during imagination of stressful or rewarding MCI compared to nonMCI, they may indicate a more general neural dysfunctioning in BPD, which could be an underlying mechanism in reaction to various stimuli. The interpretation matches reported exaggerated sensitization to emotionally salient situations, an increased experience of emotions, and dysfunctional emotion regulation abilities in BPD [13, 14]. Furthermore, it is possible that self-perception triggered by the imagination of being by oneself may be experienced as aversive by women with BPD [32, 33] and therefore, may also be associated with increased neural activity.

The AINS is part of the salience network and known to be involved in the processing of emotions [34–36]. The PINS is involved in the processing of emotional states, too, while a main focus seems to lie on the integration of sensory information [35]. Furthermore, the PINS is implicated in the remembering of interoceptive states [37] and, hence, could play a role in the imagination of the internal state during the presented scripts. In patients with BPD, the AINS and the PINS have been shown to be hyperactivated in response to negative emotional stimuli [7]. Increased AINS activity was also found in response to negative as well as positive stimuli in patients with BPD [38]. These activations might indicate a general increase in emotional sensitivity in patients with BPD (reflected in hyperactivation of the AINS). Our findings from the imagination phase showing increased bilateral insula activity point in the same direction underlining the notion of a general emotional hyperarousal across different stimuli and situations. Accordingly, in the present study mothers with BPD reported greater arousal compared to healthy mothers across all scripts. Such exaggerated sensitization to emotionally salient situations and strong experience of emotions could affect mother–child interactions by leading to an emotional over-involvement in the child’s affective state and especially the child’s distress, impeding a sensitive response to different signals of the child and mother’s (co)-regulative abilities [17, 18].

Furthermore, mothers with BPD showed increased activation of a large cluster including the dorsal and pregenual ACC during the imagination phase. As the ACC is involved in top-down regulation encompassing emotional and cognitive control [8, 39], this might indicate that hyperarousal in response to everyday situations with and without the child is associated with more regulative effort in mothers with BPD, while, ultimately, they do not seem to succeed in reducing their arousal to a level as experienced by healthy mothers. Moreover, regarding the exaggerated sensitization to salient situations and possible emotional overinvolvement, regulatory capacities might indeed be more challenged in mothers with BPD. This neural response pattern might contribute to mothers with BPD perceiving interactions with their children as more stressful and interacting more intrusively [40]. It is in line with previous results showing reduced cortisol levels in healthy mothers after mother–child interaction, while cortisol levels remained unchanged in mothers with BPD, which could indicate a lack of stress relief [5].

We then examined neural activity during the initial audio phase, in which the scripts were presented and participants may have already started imagining the situations. In this phase, we also found increased activity in the AINS in response to everyday situations in mothers with BPD compared to healthy mothers, albeit in a smaller cluster limited to the left hemisphere. Importantly, we found a significant negative correlation between activation in the left AINS and the CIB-score (a marker for the overall quality of mother–child interaction) in mothers with BPD suggesting the AINS to have relevance for parenting behavior. However, this correlation was only found in the audio phase and not in the imagination phase. Although we did not find an overall group effect regarding ACC activity in the audio phase, healthy mothers showed stronger activation of the ACC in response to MCI than nonMCI scripts, which was observed in healthy mothers only. This could indicate that healthy mothers utilize more regulative capacities during a situation of emotional involvement in stressful and rewarding situations with the child compared to nonMCI and are able to regulate their arousal when initially presented with the situation. As our paradigm does not allow to map temporal processes exactly or to directly compare the imagination and the audio phases, it should be investigated in future studies how insula and ACC activations may change over time and how such changes may implicate maternal behavior.

Mothers with BPD reported lower affect ratings in response to rMCI scripts than healthy mothers, indicating a more negative emotional perception of rMCI in mothers with BPD. However, contrary to our expectations we did not find altered neural activity in the reward system in mothers with BPD compared to healthy mothers. In previous studies, activation of the reward system in healthy mothers has been observed mainly in response to visual stimuli but not as consistently in response to auditive stimuli of the own child [17]. Hence, the type of presented stimuli could have influenced the activation of the reward system, and therefore, the present results from a script-based imagination paradigm may not be transferable to other types of stimuli. Furthermore, while comorbid psychiatric disorders might have exerted an effect on the maternal brain, the presence of comorbidities is very common in patients with BPD (e.g., [41, 42]), cannot be precisely separated from BPD psychopathology itself [41, 42], and effects of comorbid psychiatric disorders on the maternal brain cannot be meaningfully separated from the effect of BPD.

### Limitations

Despite the strengths of the present study such as the combination of neural and behavioral data in a sample of mothers with BPD with young children that has not been studied before and the use of a carefully designed fMRI paradigm, we want to acknowledge some limitations of the study. First, participants were asked to imagine mother–child interactions and situations in which the mother is alone; therefore, neural data are not available during real-life mother–child interaction. Importantly, vividness ratings of the imagination were high and did not differ between the two groups included in the study. Furthermore, there is accumulating evidence that the imagination of behavior evokes neural responses that are similar to neural responses to actual behavior, albeit using more simple stimuli than the scripts applied in the present study [43–47]. Nevertheless, the neural activation pattern observed in this study might also be influenced by different ways of achieving a vivid imagination of the situation described in the scripts and, hence, different neural avenues which cannot (yet) be addressed using our current technical possibilities. Second, although participants may have already started imagination during the audio phase, the paradigm does not allow to directly compare the imagination and the audio phases and, thus, cannot address temporal processes exactly. Third, results cannot be generalized to mothers with children in other age groups and especially not to fathers. Fathers are dramatically underrepresented in research on parenting and the parental brain and should be considered in future studies. Fourth, the results should be replicated in a bigger sample. Additionally, healthy mothers had overall high CIB values with little variability in the group, which could be causal for the missing associations in this group. Future studies should also investigate other aspects besides the processing of stress and re-ward, e.g., the hyperactivation in regions belonging to the social cognition network probably suggesting a compensatory mechanism.

## Conclusion

Results from this first study investigating the maternal brain with a focus on stress and reward in mothers with BPD indicate that an exaggerated sensitization to different, emotionally salient situations together with dysfunctional emotion regulation abilities, as reflected by increased insula and ACC activation, might hinder sensitive maternal behavior in mothers with BPD. AINS activation was associated with maternal behavior in a real-life mother–child interaction and may hence be of relevance for parenting behavior. These results underline the importance for psychotherapeutic interventions to decrease emotional hyperarousal and improve difficulties in emotion regulation in patients with BPD, especially in affected mothers caring for young children. In order to be able to target specific neural dysfunctions in BPD, future research needs to concentrate on temporal processes in mother–child interaction and should use different stimulus modalities to further elucidate neural mechanisms underlying or mediating difficulties in maternal behavior.

### Supplementary Information

Below is the link to the electronic supplementary material.

Supplementary file1 (PDF 73 KB)

## Data Availability

Due to the nature of this research, participants of this study did not agree for their data to be shared publicly, so supporting data are not available.
