# Supplementary material for: Stress and reward in the maternal brain of mothers with borderline personality disorder: a script-based fMRI study
Source: Eur Arch Psychiatry Clin Neurosci. 2023 Jun 24;274(1):117–27. doi: 10.1007/s00406-023-01634-6 (PMC10786970; doi:10.1007/s00406-023-01634-6)
Supplement: Supplementary file 1 — Supplementary file1 (PDF 73 KB) [file 406_2023_1634_MOESM1_ESM.pdf]

Supplementary material

Article title: Stress and reward in the maternal brain of mothers with borderline personality disorder - a script-based fMRI study

Journal name: European Archives of Psychiatry and Clinical Neuroscience

Authors: Isabella Schneider[a*], Sabine C. Herpertz[a], Kai Ueltzhöffer[b], Corinne Neukel[a]

[a] Department of General Psychiatry, Center for Psychosocial Medicine, Heidelberg University, Voßstr. 4, 69115 Heidelberg, Germany, [b] European Molecular Biology Laboratory, Genome Biology Unit, Meyerhofstr. 1, 69117 Heidelberg, Germany

*** Corresponding author:**

Isabella Schneider, Department of General Psychiatry

Center for Psychosocial Medicine, Heidelberg University

Voßstr. 4, 69115 Heidelberg, Germany

Isabella.Schneider@med.uni-heidelberg.de

<u>**Online Resource 1**</u>

Validation of the stimuli

Previous to the study, we undertook validation of the stimuli by having 17 healthy mothers (age: 31.6 ± 2.7 years) with at least one child (age: 2.6 ± 1.1 years; 9 boys, 8 girls) rate 18 scripts with three conditions each (rewarding mother-child interaction (rMCI), stressful mother-child interaction (sMCI), non-mother-child interaction (nonMCI)). The mothers were asked to rate the scripts on a scale of 1 - 7 according to the vividness of the situation (1=high, 7=low), as well as their affect (1=positive, 7=negative) and arousal (1=high, 7=low) when imagining the presented situation. The first criterion for selecting scripts represented experienced vividness, as imagination is central to the paradigm. Here, we selected the twelve scenes with the highest score in total for all three conditions. For the second criterion, we checked which of the twelve scripts mostly matched the profile of assigning a positive affect to a rewarding situation, a negative affect to a stressful situation, and an affect in the medium/neutral range to a non-mother-child interaction. In addition, since stressful mother child-interactions can create excitement and tension a high rating of arousal was inclusion criterion for stressful situations. Rewarding inter-actions can elicit high as well as low arousal. Therefore, we did not put a focus on arousal in the selection of rewarding scripts. Eight scenes were selected and included, which fulfilled the mentioned criteria in the best possible way. The table depicts mean values (M) and standard deviations (SD) for selected scripts.

	Affect (M ± SD)	Arousal (M ± SD)	Vividness (M ± SD)
**script 1**			
**rMCI**	1.4 ± 0.7	1.1 ± 0.3	1.8 ± 0.9
**sMCI**	6.2 ± 0.7	4.1 ± 1.7	2.0 ± 1.0
**non-MCI**	4.1 ± 1.1	3.5 ± 2.6	1.2 ± 0.6
**script 2**			
**rMCI**	1.4 ± 0.8	1.6 ± 1.1	1.6 ± 1.2
**sMCI**	6.9 ± 0.3	6.4 ± 0.8	1.8 ± 1.0
**non-MCI**	3.8 ± 0.4	1.8 ± 0.8	1.9 ± 1.3
**script 3**			
**rMCI**	1.6 ± 1.3	1.8 ± 1.3	1.8 ± 1.0
**sMCI**	6.6 ± 0.6	6.0 ± 1.3	2.1 ± 1.4
**non-MCI**	3.8 ± 1.2	3.1 ± 1.5	1.7 ± 1.0
**script 4**			
**rMCI**	1.5 ± 0.9	1.2 ± 0.4	1.6 ± 1.1
**sMCI**	6.2 ± 0.8	4.8 ± 1.4	1.4 ± 0.5
**non-MCI**	3.6 ± 0.5	1.1 ± 0.3	1.9 ± 1.4
**script 5**			
**rMCI**	1.4 ± 0.8	1.2 ± 0.4	1.4 ± 0.5
**sMCI**	6.2 ± 0.8	5.0 ± 1.2	2.0 ± 1.5
**non-MCI**	3.4 ± 1.0	1.2 ± 0.4	1.4 ± 0.9
**script 6**			
**rMCI**	1.7 ± 1.3	1.7 ± 1.3	2.0 ± 1.2
**sMCI**	6.2 ± 0.8	5.5 ± 0.9	1.9 ± 0.7
**non-MCI**	4.5 ± 0.9	2.5 ± 1.3	2.1 ± 1.0
**script 7**			
**rMCI**	1.1 ± 0.2	1.1 ± 0.3	1.2 ± 0.4

**sMCI**	6.6 ± 0.7	5.7 ± 1.4	2.4 ± 2.0
**non-MCI**	3.0 ± 0.7	1.5 ± 0.9	1.9 ± 1.4
**script 8**			
**rMCI**	1.5 ± 0.9	1.3 ± 0.5	2.0 ± 1.7
**sMCI**	6.0 ± 0.8	5.0 ± 1.1	1.9 ± 0.9
**non-MCI**	3.0 ± 1.1	1.6 ± 0.7	1.7 ± 0.7

Online Resource 2:
Scripts used in the fMRI paradigm

Scripts for mothers with daughters, corresponding scripts are also available for mothers with sons. rMCI = rewarding mother-child interaction; sMCI = stressful mother-child interaction; nonMCI = comparable situation without the child

Script 1:
rMCI: It is a beautiful and sunny day. I really want to go outside with my daughter. When I want to put on my daughter's shoes, she laughs at me and happily helps.
sMCI: It is a beautiful and sunny day. I really want to go outside with my daughter. When I want to put on my daughter's shoes, she yells at me and wants to run away.
nonMCI: It is a beautiful and sunny day. I really want to go outside today. I put on my shoes and jacket and take my bag with me. I am going outside.

Script 2:
rMCI: I am outside with my daughter. At a street, I want to hold my daughter's hand. My daughter gives me her hand and beams at me. We cross the street together.
sMCI: I am outside with my daughter. At a street, I want to hold my daughter's hand. My daughter tears her hand loose and runs away from me. I run after her into the street.
nonMCI: I am walking alone outside. I stop at a street and look around. There are no cars or bicycles coming. The street is clear. Then I cross the street quickly.

Script 3:
rMCI: I am in the supermarket with my daughter and I am in a hurry. When I want to put my daughter in the shopping cart, she lets me put her in without any problem. She looks at me with a laugh.
sMCI: I am in the supermarket with my daughter and I am in a hurry. When I want to put my daughter in the shopping cart, she angrily throws herself on the floor. She starts screaming loudly.
nonMCI: I am alone in the supermarket and I am in a hurry. I want to get a shopping cart. I untie the shopping cart and push it. The shopping cart rolls in front of me.

Script 4:
rMCI: I am at home with my daughter and we have just eaten. My daughter's hands are all sticky. When we go to wash our hands, she follows me and smiles at me.
sMCI: I am at home with my daughter and we have just eaten. My daughter's hands are all sticky. When we go to wash our hands, she runs away from me and gets everything dirty.
nonMCI: I am alone at home. I have just eaten. I take my plate and clean it up. Then I go to the sink and want to wash my hands. I turn on the water.

Script 5:
rMCI: I am at home and want to put my daughter to bed. I still want to brush her teeth. My daughter opens her mouth and lets me brush her teeth. Afterwards, she grins at me.
sMCI: I am at home and want to put my daughter to bed. I still want to brush her teeth. My daughter closes her mouth and turns away from me. She starts to scream loudly.
nonMCI: I am at home and want to go to bed. I still want to brush my teeth. I take my toothbrush and put toothpaste on it. Then I start to brush my teeth thoroughly.

Script 6:
rMCI: I am out with my daughter and I am in a hurry. We have to go up a flight of stairs quickly. When I reach out my hand to my daughter, she holds on tight. My daughter looks at me and smiles.
sMCI: I am walking with my daughter and I am in a hurry. We have to go up a flight of stairs quickly. When I reach out my hand to my daughter, she pulls her hand away and sits down. She whines.

nonMCI: I am traveling alone and am in a hurry. I have to run up a flight of stairs quickly. I hold on to the railing and walk up the stairs. Once I reach the top, I quickly continue walking.

Script 7:
rMCI: I was outside by myself and finally get back to my daughter. When she sees me, she runs up to me and stretches out her arms to me. My daughter is beaming all over.
sMCI: I was outside by myself and finally come back to my daughter. When she sees me, she runs away from me and wants to hide. My daughter starts to cry and looks away.
nonMCI: I was outside by myself and finally come home. I am alone. I open the door and take off my shoes. I put my shoes by the door. Then I take off my jacket.

Script 8:
rMCI: I am at the bakery with my daughter. My daughter really wants a pretzel. I tell her that I only want to buy bread. She is satisfied with that and smiles at me.
sMCI: I am at the bakery with my daughter. My daughter really wants to have a pretzel. I tell her that I only want to buy bread. She begins to whine loudly and gets angry.
nonMCI: I am alone at the bakery. I look at what is on display. I see different breads and pastries. I only want to buy one bread. I choose one of the breads and buy it.

Online Resource 3

Results of whole-brain analysis regarding significant group and condition differences of mothers with BPD and healthy mothers (HC) during the imagination (IP) and audio (AP) phase. r/sMCI: rewarding/stressful mother-child interaction. nonMCI: no mother-child interaction. R=right; L=left; AINS=anterior insula; DC=diencephalon; MCgG =middle cingulate cortex; MTG=middle temporal gyrus; PCgG=posterior cingulate cortex; PINS=posterior insula; STG=superior temporal gyrus; TTG=transverse temporal gyrus.

**IP**									
**Contrast**	**Cluster size (k)**	**T value**	**p value peak**	**p value cluster FWE**	**Peak voxel MNI: x y z (mm)**			**Anatomical location of peak voxel**	**Anatomical location of cluster**
BPD > HC	30	6.59	<.001	<.001	39	20	-13	R AINS	R AINS
	37	5.75	<.001	<.001	-51	-16	-16	L MTG	L MTG, L STG
	20	6.02	<.001	<.001	9	-37	2	R Thalamus	R Thalamus, R PCgG
	11	5.62	<.001	.001	45	-13	-4	R PINS	R PINS
	10	6.31	<.001	.001	-12	-13	-13	L ventral DC	L ventral DC
**AP**									
**Contrast**	**Cluster size (k)**	**T value**	**p value peak**	**p value cluster FWE**	**Peak voxel MNI: x y z (mm)**			**Anatomical location of peak voxel**	**Anatomical location of cluster**
BPD>HC	15	6.73	<.001	<.001	-45	-19	8	L TTG	L TTG
	23	6.02	<.001	<.001	51	-49	8	R MTG	R MTG
	11	5.99	<.001	.001	45	20	-10	R AINS	R AINS
rMCI > nonMCI	44	6.30	<.001	<.001	0	-19	35	L MCgG	L MCgG, R L MCgG
	11	4.99	<.001	.001	-3	-43	26	L PCgG	L PCgG
sMCI > nonMCI	22	5.74	<.001	<.001	-3	-19	35	L MCgG	L MCgG
HC: r/sMCI > nonMCI	43	6.57	<.001	<.001	0	-19	35	L MCgG	L MCgG
